# Data on pharmacological applications and hypothermia protection against in vitro oxygen-glucose-deprivation-related neurodegeneration of adult rat CA1 region

**DOI:** 10.1016/j.dib.2016.12.009

**Published:** 2016-12-15

**Authors:** Pınar Öz, Hale Saybaşılı

**Affiliations:** a Neuropsychopharmacology Application and Research Center, Üsküdar University Central Campus, Altunizade Mah. Haluk Türksoy Sk. No:14, 34662 Istanbul, Turkey; b Institute of Biomedical Engineering Boğaziçi Üniversity Kandilli Campus, Kandilli Mah., 34684 Istanbul, Turkey

**Keywords:** Oxygen-glucose-deprivation, Hippocampus, Histology

## Abstract

In this data article, the level of chemical neuroprotection against oxygen-glucose-deprivation (OGD)-related neurodegeneration in CA1 was analyzed using the measurements on CA1 stratum pyramidale (CA1sp) width. Adult rat hippocampal slices were incubated in OGD medium for 60 min to create a model for severe ischemic conditions. Alternatively, control slices were incubated in artificial cerebrospinal fluid (ACSF) for 60 min. A study of OGD induced neurodegeneration and partial prevention by pharmacological agents reported; baclofen, memantine and l-carnitine effects were included. Also, the use of hypothermia was reported (P. Öz, H. Saybaşılı, 2016) [1].

Here, the use CA1sp width measurements on Nissl-stained hippocampal slices is introduced as a valid and affordable method for detecting the level of neurodegeneration and neuroprotection on hippocampal slices. The protective effect of hypothermia was found to be more pronounced compared to other agents.

**Specifications Table**

**Table:** 

Subject area	*Biology*
More specific subject area	*Neuropharmacology*
Type of data	*Figure*
How data was acquired	*Microscopy* (Leica DM 2500, Leica Microsystems Inc., Buffalo Grove, IL, USA)
Data format	*Raw, analyzed*
Experimental factors	*Samples are taken from acute hippocampal slices, that were exposed to experimental conditions, fixed and Nissl-stained.*
Experimental features	*As an in vitro model of ischemia, rat acute hippocampal slices were exposed to 60* *min. oxygen- glucose deprivation. The neuroprotective effect of pharmacological reagents (baclofen, memantine, l-carnitine) and hypothermia was investigated by using CA1 stratum pyramidale width measurements.*
Data source location	*İstanbul, Turkey*
Data accessibility	*The data is in public repository.*

**Value of the data**

• The presented data is obtained through an easy and affordable histological method, which can be utilized to observe the morphological changes on the tissues from a broad range of *in vitro* neurodegeneration models.
• The method enables the morphological examination of hippocampal tissues that may be mainly prepared for other techniques, e.g. patch-clamp.
• The data points out the neuroprotective value of l-carnitine and hypothermia against ischemia-related neurodegeneration, in comparison with frequently used pharmacological reagents, baclofen and memantine.

## 1 Data

These data mainly focus on the dose-dependent neuroprotection provided by baclofen, memantine and l-carnitine against oxygen-glucose-deprivation (OGD)-related neurodegeneration of CA1 region, with an addition of the data obtained from the use of hypothermia instead of pharmacological agents. The data consists of CA1 stratum pyramidale (CA1sp) width measurements from slices incubated in OGD medium for 60 min with or without neuroprotective applications. The values were normalized with the CA1sp measurements of healthy slices incubated in artificial cerebrospinal fluid (ACSF) for 60 min. The dose-response curves for baclofen, memantine and l-carnitine is provided, together with the comparison of the protection levels for most effective doses of single chemical agents to chemical agent combinations or hypothermia.

## 2 Experimental design, materials and methods

Acute hippocampal slice preparation, slice incubation and histology procedures are described in [1].

**Fig. 1:**
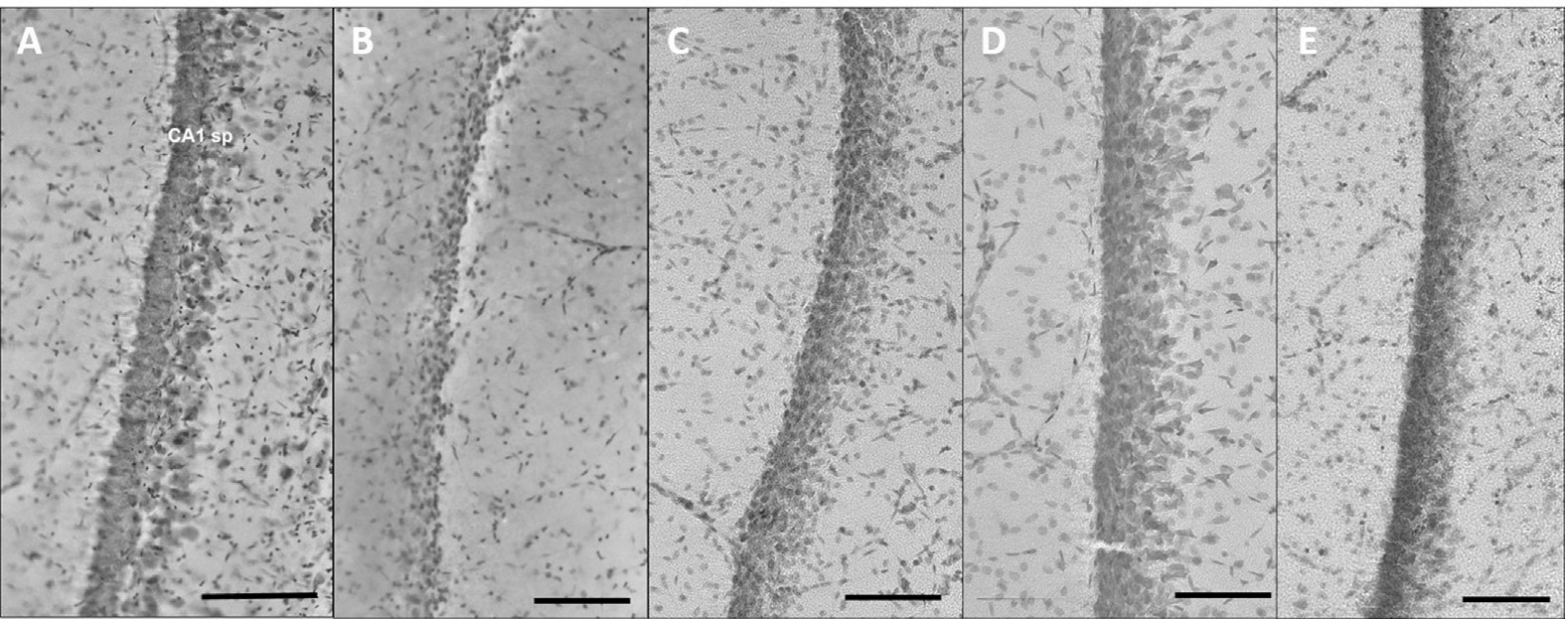
The effect of OGD-induced neurodegeneration and pharmacological neuroprotection on CA1 stratum pyramidale. The images display the CA1 stratum pyramidale at 20X magnification for (A) hippocampal slices incubated in ACSF for 60 min, (B) in OGD medium for 60 min and in OGD medium for 60 min with the addition of (C) 25 µM baclofen, (D) 25 µM memantine and (E) 25 µM l-carnitine.

The CA1sp was inspected under light microscope (Leica DM 2500, Leica Microsystems Inc., Buffalo Grove, IL, USA) and photographed with 20X magnification (DFC310FX, Leica Microsystems Inc., Buffalo Grove, IL, USA). CA1sp width measurements were carried on these images using ImageJ software [2]. The details of measurements, estimation and statistics are described in [1] (Fig. 1).

Since the CA1sp width measurements indirectly represent the health of the region, normalized CA1sp width values were given as CA1 viability percentages.

**Fig. 2:**
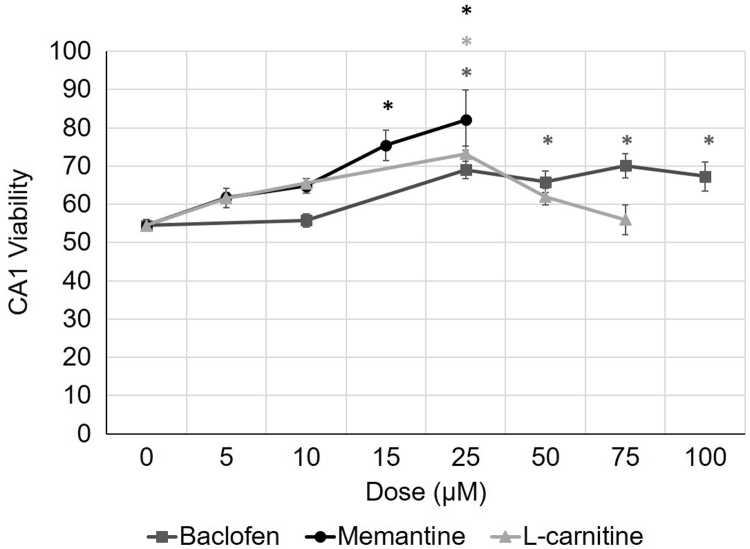
Dose-dependent neuroprotective effect of pharmacological treatment against OGD-induced damage in CA1*stratum pyramidale*. CA1sp width measurements was displayed as a measure for CA1 viability after OGD incubation. Baclofen, memantine and l-carnitine were added to OGD medium during incubation individually. All of tested pharmacological agents led to a significant increase in CA1sp width at 25 µM compared to OGD incubation without any applications (0 mM). 15 µM memantine also significantly increased CA1sp, while baclofen was only effective for 25 µM and higher doses. l-carnitine provided a neuroprotective effect only for 25 µM dose. All values were normalized with mean CA1sp width of healthy slices (ACSF incubation) and represented as percentages. *p<0.05.

Dose-response curves for baclofen (bac, Sigma-Aldrich, St. Louis, MO, USA), Memantine-HCl (mem, Sigma-Aldrich, St. Louis, MO, USA), and (L)-carnitine-HCl (lcar, Sigma-Aldrich, St. Louis, MO, USA) display the mean CA1sp width values for each dose, normalized with the mean CA1sp width from healthy control slices. Error bars represent standard errors. Zero dose represents the OGD medium incubation without any pharmacological application (negative control) (Fig. 2).

**Fig. 3:**
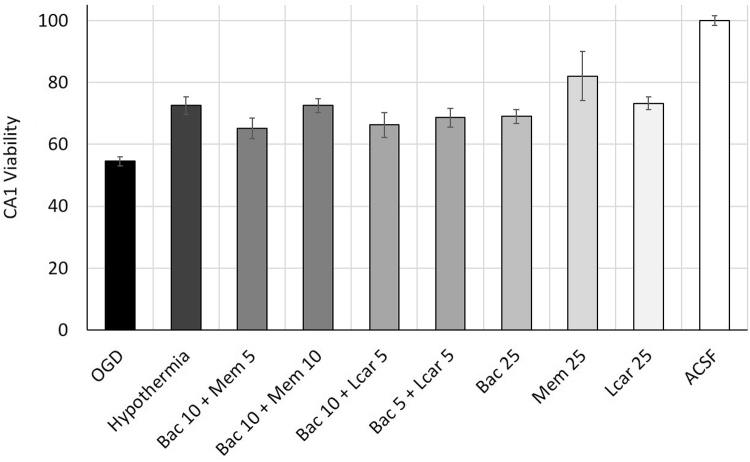
Comparison of the neuroprotective effect provided by hypothermia and combined or single applications of pharmacological agents against OGD-induced damage in CA1. CA1sp width measurements was displayed as a measure for CA1 viability after OGD incubation. Individual treatment results for 25 µM baclofen, memantine and l-carnitine represent the positive controls, while results for OGD incubation without any application and for ACSF incubation represent the negative controls. Hypothermia led to a significant increase in CA1 viability and this effect was statistically similar to the combined use of 10 µM baclofen and 10 µM Memantine and single use of 25 µM baclofen, memantine and l-carnitine. Other combination did not lead to a significant change in CA1 viability after OGD-induced damage. All values were normalized with mean CA1sp width of healthy slices (ACSF incubation) and represented as percentages. *p<0.05.

For the comparison of single pharmacological application with combinatory uses and with hypothermia, the normalized CA1sp width measurements from 25 mM bac, mem and lcar applications were represented as positive controls. The pharmacological combinations were as follows: (a) bac 10 mM+mem 5 mM, (b) bac 10 mM+mem 10 mM, (c) bac 10 mM+lcar 5 mM, (d) bac 5 mM+lcar 5 mM. All values were compared to the normalized CA1sp width measurements from slices incubated in OGD medium without any applications (negative control) and slices incubated in ACSF incubation (double negative control) (Fig. 3).

Significant differences between groups were assessed using one-way analysis of variance (ANOVA); post-hoc tests further examined between-group differences. Statistical analyses were conducted using SAS Studio 3.5 (SAS Institute Inc., Cary, NC, USA). Tukey׳s post-hoc test was used to compare individual groups amongst themselves (*α*=0.05).
